# Abnormal Temporal Coupling of Tactile Perception and Motor Action in Parkinson’s Disease

**DOI:** 10.3389/fneur.2017.00249

**Published:** 2017-06-06

**Authors:** Antonella Conte, Daniele Belvisi, Matteo Tartaglia, Francesca Natalia Cortese, Viola Baione, Emanuele Battista, Xiao Y. Zhu, Giovanni Fabbrini, Alfredo Berardelli

**Affiliations:** ^1^Department of Neurology and Psychiatry, Sapienza University Rome, Rome, Italy; ^2^IRCCS Neuromed, Pozzilli, Italy; ^3^Department of Neurology, Shanghai General Hospital, Shanghai Jiao Tong University School of Medicine, Shanghai, China

**Keywords:** Parkinson’s disease, temporal processing, somatosensory temporal discrimination threshold, movement, sensorimotor integration, basal ganglia

## Abstract

Evidence shows altered somatosensory temporal discrimination threshold (STDT) in Parkinson’s disease in comparison to normal subjects. In healthy subjects, movement execution modulates STDT values through mechanisms of sensory gating. We investigated whether STDT modulation during movement execution in patients with Parkinson’s disease differs from that in healthy subjects. In 24 patients with Parkinson’s disease and 20 healthy subjects, we tested STDT at baseline and during index finger abductions (at movement onset “0”, 100, and 200 ms thereafter). We also recorded kinematic features of index finger abductions. Fifteen out of the 24 patients were also tested ON medication. In healthy subjects, STDT increased significantly at 0, 100, and 200 ms after movement onset, whereas in patients with Parkinson’s disease in OFF therapy, it increased significantly at 0 and 100 ms but returned to baseline values at 200 ms. When patients were tested ON therapy, STDT during index finger abductions increased significantly, with a time course similar to that of healthy subjects. Differently from healthy subjects, in patients with Parkinson’s disease, the mean velocity of the finger abductions decreased according to the time lapse between movement onset and the delivery of the paired electrical stimuli for testing somatosensory temporal discrimination. In conclusion, patients with Parkinson’s disease show abnormalities in the temporal coupling between tactile information and motor outflow. Our study provides first evidence that altered temporal processing of sensory information play a role in the pathophysiology of motor symptoms in Parkinson’s disease.

## Introduction

Sensorimotor integration is the process whereby incoming sensory information is continuously monitored so as to allow the current motor plan to be adjusted and voluntary movements to be executed accurately (1–5). Sensorimotor integration depends also on the temporal processing of sensory information. In humans, one way to assess temporal processing of sensory information is to calculate the somatosensory temporal discrimination threshold (STDT)—the shortest interval at which an individual recognizes paired stimuli as separate in time (6–9). In a recent study on healthy subjects, we used the STDT to provide new evidence showing that movement execution brings about changes in the temporal processing of tactile information through the interplay between the basal ganglia and thalamus (10).

Several studies have consistently reported abnormally high STDT values in patients with Parkinson’s disease (6, 11–15). STDT abnormalities in PD patients parallel disease severity and duration and are partially improved by dopaminergic medication (6, 11–16). Whether STDT modulation during movement execution in PD is abnormal and whether any abnormalities in STDT modulation contribute to motor symptoms in PD is unclear. Knowing more about tactile information during movement execution would increase our understanding of the pathophysiology of motor symptoms in PD.

The aim of this study was to investigate whether STDT modulation during movement execution in PD patients differs from that in healthy subjects and to evaluate changes in the kinematic properties of movements during the sensorimotor integration task. We also investigated the effects of dopaminergic therapy on movement-induced STDT modulation. Finally, we investigated possible correlations between neurophysiological and clinical variables.

## Materials and Methods

We enrolled 24 patients with Parkinson’s disease (15M/9F; aged 61 ± 8 years) and 20 age-matched healthy subjects (13M/7F; aged 59 ± 9 years) at the Department of Neurology and Psychiatry of the University of Rome, Sapienza. All the participants gave their written informed consent. The experimental procedure was approved by the institutional review board at Sapienza University Rome and conducted in accordance with the Declaration of Helsinki. The diagnosis of PD was based on clinical criteria (17, 18). Disease severity was scored using the Hoehn and Yahr scale and the MDS-Unified Parkinson’s Disease Rating Scale part III (MDS-UPDRS part III). Patients were also evaluated by means of the Frontal Assessment Battery (FAB) and the Montreal Cognitive Assessment (MOCA) (Table 1). Since data yielded by STDT testing are only reliable if there is no clear deficit in cognitive functions, patients with a FAB score lower than 15 and a MOCA score lower than 24 were excluded. Subjects who had a clinically diagnosed peripheral sensory neuropathy were also excluded.

**Table 1:** Clinical and demographic features of patients with Parkinson’s disease.

Patient	Disease duration (years)	Hoehn and Yahr	UPDR 1S ON	UPDRS OFF	Frontal Assessment Battery	Montreal Cognitive Assessment
1	5	3	12	20	16	27
2	9	1.5	11	18	17	27
3	4	2	8	14	17	25
4	6	2	15	23	16	27
5	2	2	13	20	17	27
6	3	2	12	20	15	26
7	6	2	10	15	16	25
8	1	1.5	14	20	17	28
9	4	1	8	12	16	28
10	3	2	14	22	16	28
11	5	3	16	26	16	28
12	4	3	14	19	17	29
13	3	2.5	7	12	17	28
14	1	3	22	30	15	25
15	7	2	14	23	17	29
16	1	1.5	−	10	16	28
17	4	1	−	16	15	27
18	9	2	−	21	16	28
19	2	2	−	22	18	27
20	4	2.5	–	43	15	26
21	4	2	–	19	16	28
22	3	2	–	21	16	26
23	3	2	–	19	17	27
24	1	1.5	–	12	18	29
Mean	3.9	2.1	12.6	19.8	16.3	27.2
SD	2.2	0.5	3.7	6.9	0.8	1.4

All the PD patients were studied OFF therapy (at least 12 h after the last dose of oral dopaminergic therapy). Fifteen of the 24 PD patients were also tested in “ON” state after receiving levodopa; the two sessions in these patients were performed in a randomized order, at least 1 week apart.

### STDT Testing

STDT was investigated on the volar surface of the index finger of the right hand and of the left hand in patients and of the right hand in healthy subjects, according to the experimental procedures previously used (7, 14, 19, 20). Participants were comfortably seated in an armchair beside a table. We delivered paired stimuli starting with an interstimulus interval (ISI) of 0 ms (simultaneous pair), and progressively increased the ISI in 10-ms steps with a staircase method. Paired tactile stimuli consisted of square-wave electrical pulses delivered using a constant current stimulator (Digitimer DS7AH) through surface skin electrodes with the anode located 0.5 cm distally to the cathode. Stimulation intensity was defined for each subject by delivering a series of stimuli at increasing intensities from 2 in 0.5 mA steps; the intensity used for STDT testing was the minimal intensity the subject perceived in 10 out of 10 consecutive stimuli. The first of three consecutive ISIs at which participants recognized the stimuli as temporally separate was considered the STDT. To keep the subjects’ attention level constant during the test and minimize possible perseverative responses, we included “catch” trials consisting of a single stimulus delivered randomly.

### Electromyographic Recordings

EMG activity was recorded through surface electrodes placed over the FDI muscle, in a belly tendon configuration. EMG signals were recorded and filtered with a Digitimer D360 (Digitimer Ltd., UK) (bandwidth 20 Hz–1 kHz), then analyzed off-line with a personal computer through a 1401 plus A/D laboratory interface (Cambridge Electronic Design, UK). Data were stored on a laboratory computer for on-line visual display and further off-line analysis (Signal software; Cambridge Electronic Design).

### Kinematic Recording

The SMART analyzer motion system (BTS Engineering, Milan, Italy), equipped with three infrared cameras (sampling rate, 120 Hz), was used to record index finger abductions. The arm was abducted at the shoulder by about 45–50°, and the elbow joint was flexed at about 90°. An optical marker was placed over the distal phalanx of the index finger. After a verbal “go” signal, subjects abducted the index finger, then returned the finger to the starting position upon being given a verbal “stop” signal shortly after (21–23). Marker displacement was reconstructed *via* a dedicated software that runs the automatic algorithm to compute the range of motion (ROM), which represents the displacement of the index finger around its metacarpophalangeal joint expressed as the degree of the angle and mean velocity (degrees per second) (BTS Engineering, Milan, Italy).

### Experimental Design

Experimental procedures envisaged first the assessment of basal STDT values (without movement). Participants were then instructed to perform index finger movements. Subjects were asked to abduct the index finger as widely and as quickly as possible and were continuously encouraged to do so throughout the motor task. Participants performed 10 index finger abductions before starting the experiment to familiarize with the motor task required (data not entered in the statistical analysis). The experimental tasks consisted of index finger abductions, with the STDT being tested on the volar surface of the same index finger. Paired stimuli for STDT were triggered by movement execution at various time lapses after movement onset. The threshold to identify movement onset of index finger abductions was set at 100 μV of EMG activity. To define the time course of movement-induced STDT changes, paired stimuli were delivered as soon as the EMG signals reached 100 μV in amplitude (defined as “0 ms” for simplicity), 100 and 200 ms after movement onset. The STDT values were thus calculated for each time lapse (0, 100, and 200 ms). At each index finger abduction, the ISI for STDT testing was progressively increased in 10-ms steps, until the subject recognized the two stimuli as sequential (10). Blocks for each time lapse were delivered in random order.

### Statistical Analysis

We first checked STDT and kinematic data for normal distribution using Shapiro–Wilk test. Having excluded any assumption violations, we then performed separate between-group ANOVA to compare baseline absolute values of STDT and kinematic values (ROM and mean velocity) in healthy subjects and patients with Parkinson’s disease. To evaluate any difference in the amount of STDT modulation and changes in kinematic variables during movement execution between patients and healthy subjects, we used separate between-group repeated measure ANOVA with factor GROUP and factor TIME LAPSE (STDT: four levels: baseline, 0, 100, and 200 ms; kinematic variables: movement only, 0, 100, and 200 ms) entering percentage values. To evaluate changes in the STDT modulation and kinematic variables induced by dopaminergic therapy, we used separate repeated measure ANOVA with factor THERAPY (two levels: ON and OFF), SIDE (more and less affected hand), and TIME LAPSE (STDT: four levels: baseline, 0, 100, and 200 ms; kinematic variables: movement only, 0, 100, and 200 ms). As a *post hoc* analysis, we used the paired sample *T* test to compare values at the different time points from baseline. Finally, we used Spearman’s correlation coefficient to evaluate possible correlations between clinical/demographic (age, sex, disease duration, age at onset, UPDRS part III scores, and upper limb bradykinesia scores) and neurophysiological results. *P* < 0.05 indicated statistical significance.

## Results

PD patients and healthy subjects did not differ in terms of age (unpaired sample *T* test: *P* = 0.43) and sex (Mann–Whitney *U* test: *P* = 0.77).

### Changes in STDT Values during Movement Execution in PD Patients and Healthy Subjects

**Figure 1:**
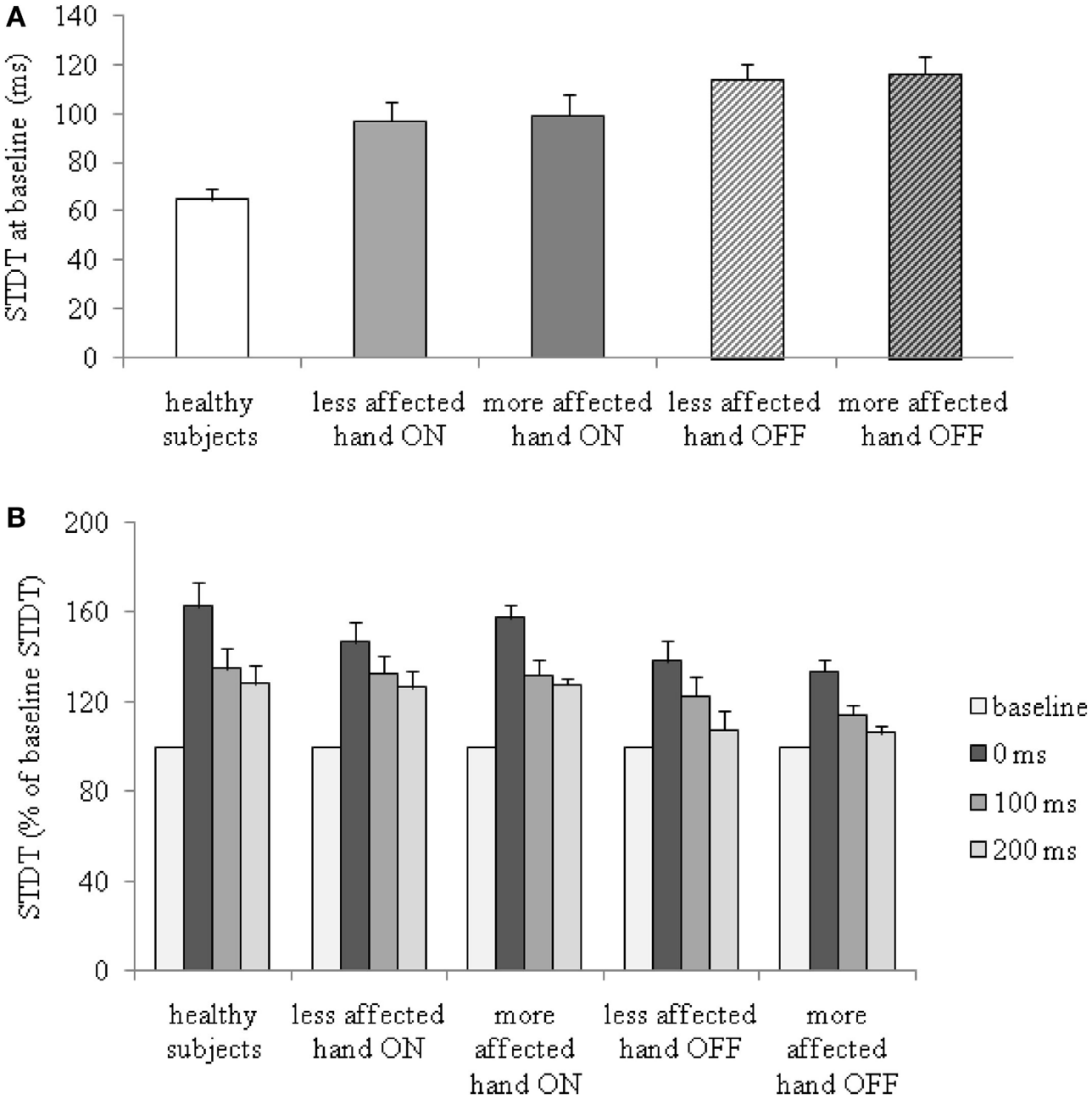
Changes in somatosensory temporal discrimination threshold (STDT) during index finger abductions in patients with Parkinson’s disease, in the ON and OFF therapy conditions, and in healthy subjects. **(A)** STDT at baseline (absolute values); **(B)** percentage changes of STDT during index finger abductions.

Between-group ANOVA for the STDT values at baseline in PD patients and healthy subjects showed that STDT values for both hands of PD patients OFF therapy [more affected hand: *F*_(1,42)_ = 26.4, *P* < 0.0001; less affected hand: *F*_(1,42)_ = 32.0, *P* < 0.001] as well as ON therapy [more affected hand: *F*_(1,33)_ = 10.5, *P* = 0.003, less affected hand: *F*_(1,33)_ = 11.5, *P* = 0.002] were higher than in healthy subjects. No significant difference was observed in the baseline STDT values of PD patients between the more affected and less affected hand (OFF: *P* = 0.5; ON: *P* = 0.73) (Figure 1A).

Index finger abductions significantly increased STDT values in PD patients in the OFF and ON therapy conditions in both hands and in healthy subjects. Between-group ANOVA performed to compare changes in STDT values during index finger abductions in patients OFF therapy and healthy subjects revealed a significant factor GROUP [more affected hand OFF therapy: *F*_(1,42)_ = 7.8, *P* = 0.008; less affected hand OFF therapy: *F*_(1,42)_ = 4.5, *P* = 0.03], factor TIME LAPSE [more affected OFF therapy: *F*_(3,126)_ = 40.6, *P* < 0.000001; less affected OFF therapy: *F*_(3,126)_ = 37.4, *P* < 0.0001], and a significant GROUP × TIME LAPSE interaction [more affected OFF therapy: *F*_(3,126)_ = 4.0, *P* = 0.009; less affected OFF therapy: *F*_(3,126)=_2.7, *P* = 0.048]. Between-group ANOVA to compare changes in STDT values during index finger abductions in patients ON therapy and healthy subjects showed a significant factor TIME LAPSE [more affected hand ON therapy: *F*_(3,99)_ = 33.9, *P* < 0.0001; less affected hand ON therapy: *F*_(3,99)_ = 32.3, *P* < 0.0001] but not significant GROUP × TIME LAPSE interaction. *Post hoc* analysis showed that the increase in STDT values in healthy subjects was significant at 0 ms (*P* < 0.0001), 100 ms (*P* = 0.001), and 200 ms (*P* = 0.002). In PD patients in the OFF therapy condition, the STDT increased at 0 ms (more affected hand: *P* < 0.0001, less affected hand: *P* < 0.0001) and 100 ms (more affected hand: *P* = 0.004, less affected hand: *P* = 0.002) but returned to baseline values at 200 ms (more affected hand: *P* = 0.07, less affected hand: *P* = 0.07). In PD patients ON therapy, the STDT increased significantly at 0 ms (more affected hand: *P* < 0.0001, less affected hand: *P* < 0.0001), 100 ms (more affected: *P* = 0.002, less affected: *P* = 0.001), and 200 ms (more affected: *P* = 0.006, less affected: *P* = 0.004) (Figure 1B). Repeated measures ANOVA performed to evaluate the effects of dopaminergic therapy on changes in the STDT values during index finger abductions in PD patients showed a significant factor THERAPY [*F*_(1,14)_ = 9.8, *P* = 0.007], factor TIME LAPSE [*F*_(3,42)_ = 26.5, *P* < 0.001] and a significant THERAPY × TIME LAPSE interaction [*F*_(3,42)_ = 2.7, *P* = 0.04]. STDT values in PD patients were significantly higher OFF therapy than ON therapy (Figure 1A). The time course related to movement onset associated with the increase in STDT values during index finger abductions in PD patients was shorter than that observed in healthy subjects when patients were tested OFF therapy (increase in STDT was significant at 0 and 100 ms interval but not at 200 ms), whereas it was similar to that observed in healthy subjects (increase in STDT was significant at 0, 100, and 200 ms interval) when tested ON therapy (Figure 1B).

### Changes in Kinematic Features of Movements Induced by STDT Testing in PD Patients and Healthy Subjects

**Figure 2:**
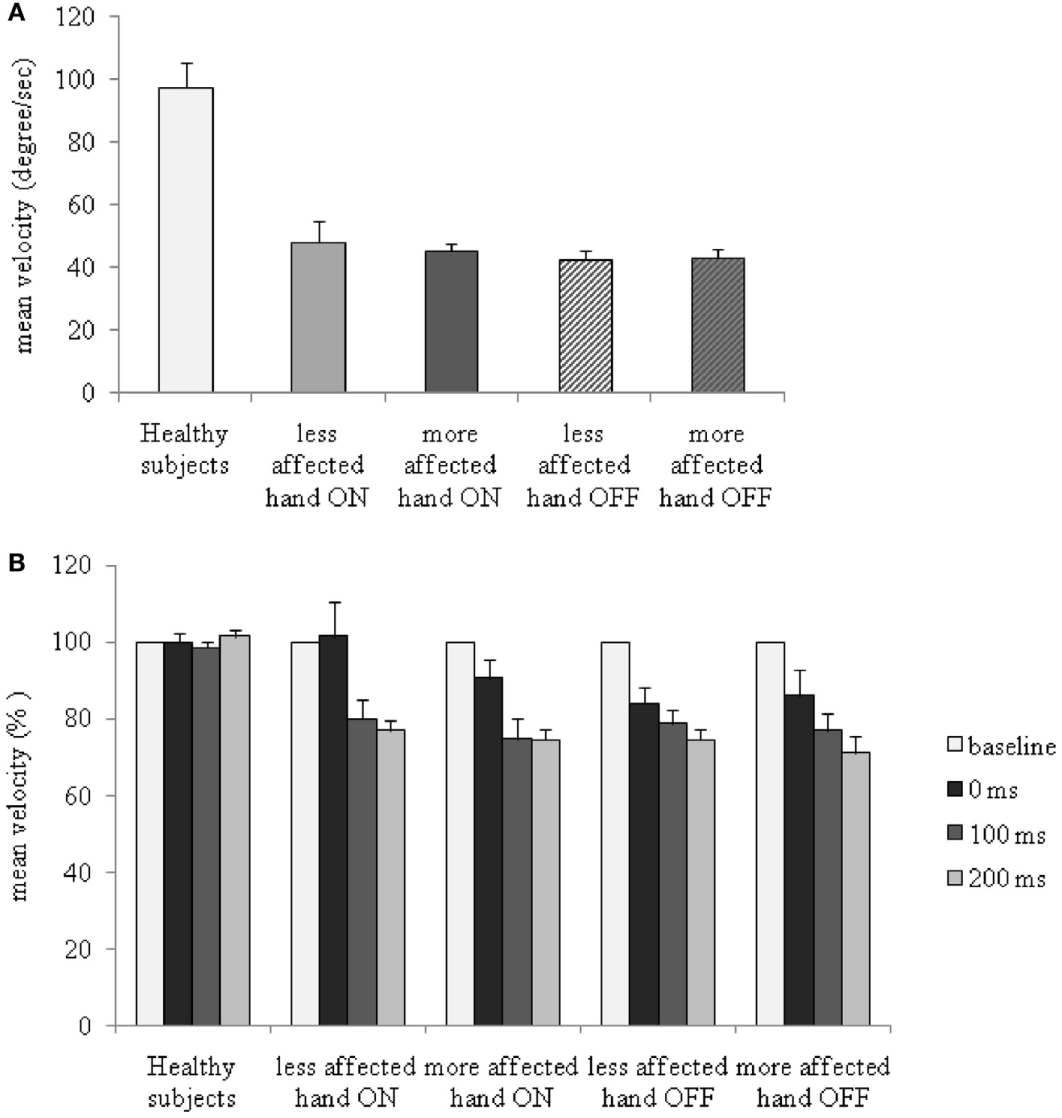
Changes in mean velocity of index finger abductions without somatosensory temporal discrimination threshold (STDT) and during STDT testing at different intervals from movement onset in patients with Parkinson’s disease, ON and OFF therapy, and in healthy subjects. **(A)** Mean velocity (absolute values) of index finger abductions without STDT testing. **(B)** *Y* axis: percentage changes in mean velocity.

Between-group ANOVA performed to compare baseline ROM and mean velocity of index finger abductions in PD patients OFF and ON therapy and healthy subjects revealed a significant factor GROUP [ROM: more affected hand OFF therapy: *F*_(1,42)_ = 7.7, *P* = 0.008; less affected hand OFF therapy: *F*_(1,42)_ = 14.0, *P* = 0.0001; more affected hand ON therapy: *F*_(1,33)_ = 14.3; *P* < 0.001; less affected hand ON therapy: *F*_(1,33)_ = 6.8, *P* = 0.01; mean velocity: more affected hand OFF therapy: *F*_(1,42)_ = 27.2, *P* < 0.001; less affected hand OFF therapy: *F*_(1,42)_ = 24.9, *P* < 0.0001; more affected hand ON therapy: *F*_(1,33)_ = 15.2, *P* = 0.001; less affected hand ON therapy: *F*_(1,33)_ = 14.4, *P* = 0.001] (Figure 2A). ROM and mean velocity in PD patients were generally lower than that in healthy subjects in the OFF and ON therapy and in more affected and less affected hands.

Between-group repeated measure ANOVA to compare percentage changes in ROM during index finger abductions in PD patients and in healthy subjects across the various time lapses tested showed a significant factor GROUP [only for more affected hand OFF: *F*_(1,42)_ = 4.7, *P* = 0.03], but no significant factor TIME LAPSE (all *P*s > 0.05) or TIME LAPSE × GROUP interaction (all *P*s > 0.05).

Between-group repeated measure ANOVA performed to compare changes in mean velocity of index finger abductions in PD patients OFF and ON therapy and healthy subjects revealed a significant factor GROUP [more affected hand OFF therapy: *F*_(1,42)_ = 24.8, *P* < 0.001; less affected hand OFF therapy: *F*_(1,42)_ = 91.0, *P* < 0.0001; more affected hand ON therapy: *F*_(1,33)_ = 99.1, *P* < 0.0001; less affected hand ON therapy: *F*_(1,33)_ = 16.3, *P* < 0.001], factor TIME LAPSE [more affected hand OFF therapy: *F*_(3,126)_ = 7.4, *P* = 0.0001; less affected hand OFF therapy: *F*_(3,126)_ = 7.9, *P* < 0.0001; more affected hand ON therapy: *F*_(3,99)_ = 10.7, *P* = 0.0001; less affected hand ON therapy: *F*_(3,99)_ = 6.1, *P* = 0.001], and a significant GROUP × TIME LAPSE interaction [more affected hand OFF therapy: *F*_(3,126)_ = 8.5, *P* < 0.001; less affected hand OFF therapy: *F*_(3,126)_ = 8.4, *P* = 0.001; more affected hand ON therapy: *F*_(3,99)_ = 10.8, *P* < 0.001; less affected hand ON therapy: *F*_(3,99)_ = 6.02, *P* = 0.001]. Index finger abductions were slower in both hands in PD patients OFF therapy than either in those ON therapy or in healthy subjects at all the time lapses tested (movement only, 0, 100, and 200 ms interval). *Post hoc* analysis performed to evaluate percentage changes in the mean velocity of index finger abductions during STDT testing showed that the mean velocity in PD patients, unlike that in healthy subjects (in whom the mean velocity was not affected by STDT testing) (*P* > 0.05), significantly decreased across time lapses, with lower values being recorded at 200 ms (more affected hand OFF therapy: *P* < 0.001; less affected hand OFF therapy: *P* < 0.001; more affected hand ON therapy: *P* < 0.001; less affected hand ON therapy: *P* = 0.001) than at 0 ms interval (Figure 2B).

ANOVA performed to analyze changes in ROM induced by dopaminergic therapy in PD patients revealed a significant HAND × THERAPY × TIME LAPSE interaction [*F*_(3,42)_ = 7.4, *P* = 0.001]. When PD patients were tested in OFF therapy condition ROM slightly decreased across ISIs, being smaller at 200 ms interval in comparison to 0 ms interval, whereas, when PD patients were tested in the ON therapy condition, similar to healthy subjects, ROM remained unchanged across time lapses.

ANOVA performed to assess the changes in mean velocity of index finger abductions of the more affected and less affected hands in PD patients ON and OFF therapy disclosed a significant factor TIME LAPSE [*F*_(3,42)_ = 38.8, *P* < 0.0001], and a significant TIME LAPSE × HAND × THERAPY interaction [*F*_(3,42)_ = 5.56, *P* = 0.002]. In PD patients, dopaminergic therapy increased the mean velocity in both the less affected and the more affected hand. The increase in mean velocity induced by dopaminergic medication was more evident at the 0 ms than at 200 ms interval, as well as in the less affected hand than in the more affected hand (changes in mean velocity in comparison to baseline values: less affected hand ON 0 ms interval: *P* = 0.96; 200 ms interval: *P* < 0.01; less affected hand OFF 0 ms interval: *P* < 0.0001; 200 ms interval: *P* < 0.00001; more affected hand ON: 0 ms interval: *P* = 0.02; 200 ms interval: *P* < 0.001; more affected hand OFF: 0 ms interval: *P* = 0.007; 200 ms interval: *P* < 0.00001) (Figure 2B). Dopaminergic therapy therefore increased the velocity of index finger abductions during STDT testing when paired electrical stimuli were given concomitantly with movement onset, but improved the mean velocity to a lesser extent when STDT was tested during the ongoing phase of the movement (200 ms interval).

**Figure 3:**
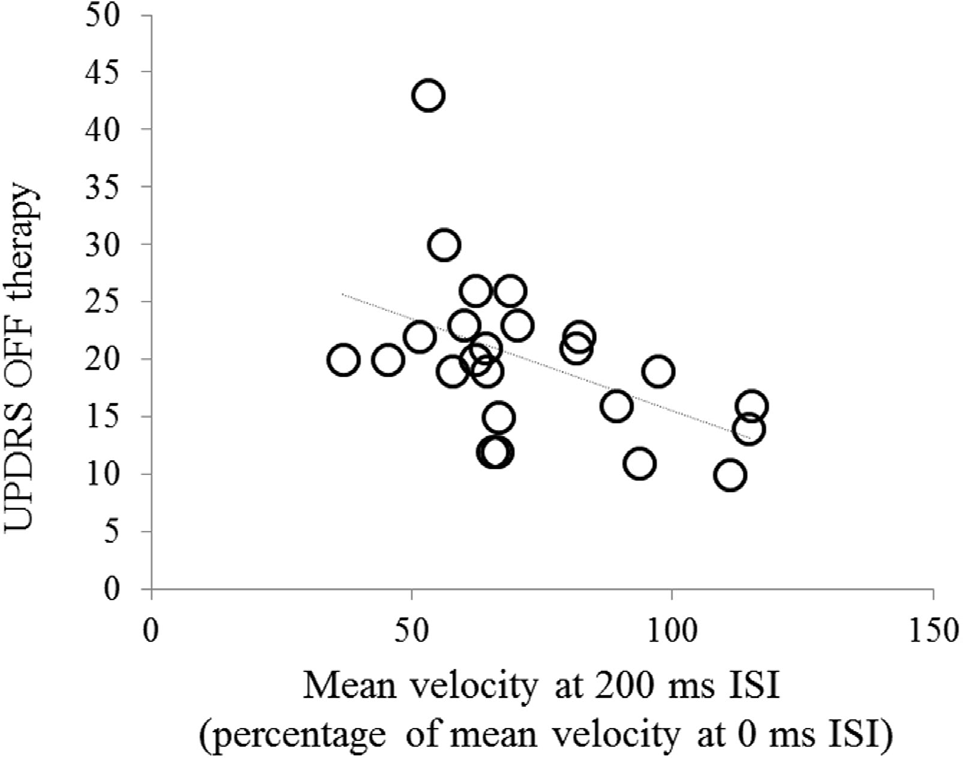
Correlation between changes in mean velocity [mean velocity of index finger abductions in the 200 ms interstimulus interval (ISI) trial expressed as percentage of mean velocity at the 0 ms ISI trial] in the more affected hand and UPDRS part III score in the OFF therapy condition.

Changes in mean velocity in the more affected hand OFF therapy at 200 ms interval were inversely related to the UPDRS part III score OFF therapy (*r* = −0.52, *P* = 0.008) (Figure 3). We did not observe any other significant relationship between the neurophysiological findings and demographic and clinical data (age, sex, age at onset, and disease duration). We only found a trend toward significance for relationship between changes in mean velocity in the more affected hand OFF therapy at 200 ms interval and the more affected hand bradykinesia scores (*r* = −0.49, *P* = 0.08).

## Discussion

The results of this study first confirm that STDT values are higher in PD patients than in healthy subjects (6, 11–16, 19). The novel finding of our study is that when the STDT was tested during index finger movements, in healthy subjects it increased significantly at 0, 100, and 200 ms intervals after movement onset, whereas in PD patients in the OFF therapy condition, it increased significantly at 0 and 100 ms but returned to baseline values at 200 ms. When PD patients were tested under dopaminergic medication, the STDT values during index finger abductions increased significantly, with a time course similar to that of healthy subjects. The other novel finding of our study was that while STDT testing during index finger abductions in healthy subjects did not result in any changes in the movement kinematics, in PD patients the mean velocity of the finger abductions decreased according to the time lapse between movement onset and the delivery of the paired electrical stimuli for STDT testing. Finally, dopaminergic medication increased the mean velocity of index finger abductions during STDT testing when paired electrical stimuli were delivered in the initial phase of movement, but failed to improve movement performance when the paired electrical stimuli were delivered during the ongoing movement.

Since we randomized the order of the trials testing the different time lapses between movement onset and paired stimuli for STDT, we are confident that changes in the STDT values and in movement kinematics did not depend on different attention levels or fatigue. Similarly, we alternated the order of the OFF and ON medication conditions. Thus, since the time lapses and medication conditions were randomized, the changes we observed in movement kinematics at different time lapses suggest that they were due specifically to the time lapse between movement onset and paired electrical stimuli. We also rule out that the time course of the STDT changes during index finger abductions in the OFF therapy condition was due to the higher baseline STDT values in PD patients because the STDT increased significantly at 0 and 100 ms. Moreover, with the stepwise method, we used for STDT, a floor effect might occur in experimental protocols in which STDT values are expected to decrease below the threshold, whereas values may increase to a considerable extent above the threshold.

The distinctive finding of our study is that temporal coupling of tactile sensory information processing and motor outflow is altered in PD patients, which extends our knowledge by bridging the gap between altered temporal processing of sensory information and motor symptoms. We show that sensory information processing related to STDT is abnormally gated during movement execution in PD patients OFF therapy if compared with healthy subjects and those ON therapy. Using our time-controlled experimental paradigm, we now provide evidence that tactile information gating is less efficient in PD patients than in healthy subjects, as indicated by the observation that STDT values of PD patients OFF therapy returned to those at baseline when STDT was tested 200 ms after movement onset, whereas the STDT was still gated in healthy subjects at this time interval. We therefore hypothesize that dopaminergic depletion in PD not only affects sensory processing in the temporal domain but also alters the temporal coupling between tactile information and motor outflow. Consistent with previous observations (6, 16, 19, 24) dopamine improved although not normalized STDT abnormalities. l-DOPA likely improves the time-related interaction between corticosubcortical structures (9, 16, 19) but fails to intervene in the dysfunctional non-dopaminergic mechanisms (14, 24–27). The novel finding of our study is that when PD patients were tested under dopaminergic therapy the STDT modulation during movement execution had a time course similar to that observed in healthy subjects. Thus, the normalized STDT modulation in PD patients in the ON medication condition suggests that abnormal sensorimotor integration we tested reflects dopaminergic loss and the basal ganglia contribution in this task.

In keeping with previous studies (28–30), index finger abductions in our PD patients were slower and of lower amplitude than those obtained from healthy subjects. We also confirm that dopaminergic therapy improves the mean velocity of index finger abductions (24, 31). The unexpected novel finding of our study is that kinematic features of index finger abductions in PD patients, unlike healthy subjects, changed according to the time lapse between paired electrical stimuli and movement onset, with slower values being observed when 200 ms elapsed between movement onset and paired stimuli for STDT than when paired stimuli were delivered concomitantly with movement onset. Since the performance of PD patients worsens during dual task procedures (32–34), one explanation for the decrease in the mean velocity of index finger abductions during STDT testing in PD patients might be the dual task design of our experimental protocol. However, we can exclude this hypothesis since changes in the mean velocity of index finger abductions were significant only at 200 ms all the intervals tested implied a dual task effect. The time interval between the paired stimuli and movement onset thus seems to play a crucial role in changes in movement kinematics and in STDT gating. We may speculate that when STDT processing is not properly gated (as suggested by the observation that STDT values returned to baseline in the OFF therapy condition at 200 ms interval), movement performance deteriorates in terms of velocity. Since in PD sensory gating alterations in the temporal domain parallel disease severity (14), we hypothesize that the more altered temporal sensory processing is, the worse the effect of abnormal sensory gating on sensorimotor integration. This hypothesis is supported to some extent by the inverse relationship we found between the STDT-induced decrease in mean velocity at 200 ms interval and the UPDRS score in the OFF therapy assessment. Whereas previous studies have provided indirect evidence showing that STDT abnormalities may contribute to impaired finger dexterity in PD (15, 35) we now show that altered temporal processing of tactile information affects movement execution by inducing abnormal temporal coupling of the two systems. Why sensorimotor integration unexpectedly fails when the movement is ongoing and not at movement onset deserves a comment. Since both patients and healthy subjects were blind to the time intervals between movement onset and paired stimuli for STDT and since the order of the time intervals was randomized, we can rule out the possibility that subjects expected the paired stimuli and, as they perceived no stimuli immediately after movement onset, the velocity of index finger movements consequently slowed down. If this were the case, the mean velocity would have decreased at the 200 ms interval even in healthy subjects. In addition, the finding that mean velocity did not vary in healthy subjects and decreased at 200 ms in patients regardless of the order of presentation of the time lapses goes against the presence of an aspecific expectation-related effect. An animal model of PD has shown that dopamine depletion reduces the level of selectivity of information that passes through the basal ganglia–cortical loops (36, 37), with a reduced signal-to-noise ratio resulting in a selection deficit between high- and low-priority information in movement execution. The consequent information overflow back to the cortex may alter the motor programs needed to adapt movement to context. The hypothesis which in our opinion best explains the decreased mean velocity at the 200 ms interval in PD patients is that, following the loss of selectivity in the basal ganglia–thalamus interplay (16, 37, 38), the motor program underlying movement execution becomes more susceptible to interference when it is ongoing and not sufficiently energized. Dopaminergic depletion reduces the ability of basal ganglia to remove inhibitory influences from the desired motor output, which normally allows the movement to proceed when already initiated (38). In this view, our experimental protocol implies that the electrical stimuli for STDT testing at 0 and 100 ms intervals are processed in the initial phase of movement, whereas at 200 ms interval they are processed during the ongoing movement. Alternative to a specific alteration of tactile–motor integration, we may hypothesize that 200 ms time lapse between movement onset and electrical stimuli for STDT may be a time window that makes the ongoing movement susceptible to a distractive effect induced by electrical stimulation. Transient attention can dynamically modulate perception when an unexpected stimulus is presented (39) and event-related potentials studies have shown that transient attention dynamically modulate SEP components beyond 100 ms (39). Our experimental protocol, however, implied that subjects were aware of the electrical stimuli in the task, insofar electrical stimuli were unlikely unexpected. Future studies in PD patients investigating whether ongoing movement is susceptible to external interference also with other sensory modalities may clarify whether changes in movement velocity during sensorimotor integration relies on attention-mediated processes.

In conclusion, altered tactile–motor integration, in addition to the previously reported proprioceptive–motor integration (15, 16, 35, 40–44), may contribute to the development of movement deficits in Parkinson’s disease. Our findings also show that dopaminergic therapy normalizes STDT gating and to some extent compensates for the decreased velocity of index finger abductions at 200 ms interval. Finally, we extend the significance of our findings to the clinical field by suggesting that studies aimed at developing devices designed to improve motor disturbances in PD patients, through mechanisms of sensorimotor integration, should take into account the precise timing of the external sensory cue in relation to the movement phase. Future confirmatory studies manipulating sensory information processing and motor behavior in PD are needed to definitively clarify the link between the abnormal temporal processing of sensory information and the pathophysiology of specific motor disturbances in PD.

## Ethics Statement

All the participants gave their written informed consent. The experimental procedure was approved by the institutional review board at Sapienza University Rome and conducted in accordance with the Declaration of Helsinki.

## Author Contributions

AC and DB: conception/design/execution of the project/drafting the manuscript. MT, NC, VB, EB, and XZ: acquisition of data/data analysis. GF and AB: revising the work critically for important intellectual content. All authors: manuscript preparation.

## Disclaimer

AC received funds from Sapienza University of Rome for a research project on movement disorders. DB, MT, NC, VB, EB, and XZ reported no disclosure. GF received funds from Sapienza University of Rome for research projects on movement disorders. AB received funds from the Benign Essential Blepharospasm Research Foundation for research on blepharospasm. He has received national grants from Italian Ministry of University and Sapienza University of Rome, Chiesi, Lundbeck, Merz, Allergan, Ipsen. He has received honoraria for lecturing from Boehringer Ingelheim, GSK Pharmaceutical, Novartis Pharmaceuticals, Lundbeck, Chiesi.

## Conflict of Interest Statement

The authors declare that the research was conducted in the absence of any commercial or financial relationships that could be construed as a potential conflict of interest.
